# Case-Control Study of the Etiology of Infant Diarrheal Disease in 14 Districts in Madagascar

**DOI:** 10.1371/journal.pone.0044533

**Published:** 2012-09-17

**Authors:** Rindra Randremanana, Frédérique Randrianirina, Marie Gousseff, Natasha Dubois, Richter Razafindratsimandresy, Elisoa Ratsima Hariniana, Benoit Garin, Arthur Randriamanantena, Hanitra Clara Rakotonirina, Lovasoa Ramparany, Charles Emile Ramarokoto, Fanjasoa Rakotomanana, Maherisoa Ratsitorahina, Soatiana Rajatonirina, Antoine Talarmin, Vincent Richard

**Affiliations:** 1 Epidemiologic Unit, Institut Pasteur in Madagascar, Antananarivo, Madagascar; 2 Clinical Biology Center, Institut Pasteur in Madagascar, Antananarivo, Madagascar; 3 Virological Unit, Institut Pasteur in Madagascar, Antananarivo, Madagascar; 4 Molecular Biology Unit, Institut Pasteur in Madagascar, Antananarivo, Madagascar; 5 Institut Pasteur in Madagascar, Antananarivo, Madagascar; National Institutes of Health, United States of America

## Abstract

**Background:**

Acute diarrhea is a major cause of childhood morbidity and mortality worldwide. Its microbiological causes and clinico-epidemiological aspects were examined during the rainy seasons from 2008 to 2009 in 14 districts in Madagascar.

**Methods:**

Stool specimens of 2196 children with acute diarrhea and 496 healthy children were collected in a community setting. Intestinal parasites were diagnosed by microscopy and bacteria by culturing methods. Rota-, astro and adenoviruses were identified using commercially available ELISA kits and rotaviruses were confirmed using reverse transcriptase polymerase chain reaction (RT-PCR).

**Results:**

Intestinal microorganisms were isolated from 54.6% of diarrheal patients and 45.9% of healthy subjects (p = <0.01). The most common pathogens in diarrheic patients were intestinal parasites (36.5%). Campylobacter spp. and Rotavirus were detected in 9.7% and 6.7% of diarrheic patients. The detection rates of *Entamoeba histolytica*, *Trichomonas intestinalis* and *Giardia lamblia* were much greater in diarrheal patients than in non diarrheal subjects (odds ratios of 5.1, 3.2, 1.7 respectively). The abundance of other enteropathogens among the non diarrheal group may indicate prolonged excretion or limited pathogenicity.

**Conclusion:**

In developing countries, where the lack of laboratory capacities is great, cross sectional studies of enteropathogens and their spatial distribution, including diarrheal and non diarrheal subjects, are interesting tools in order to advise regional policies on treatment and diarrheic patient management.

## Introduction

Diarrheal diseases are a major public health problem that particularly affect children in developing countries [1], with over 1.8 million children under 5 years of age dying of diarrheal disease each year [2].

Because childhood mortality worldwide occurs more often in African countries [2], it is necessary to gain knowledge on the etiologic and epidemiologic features of diarrheal diseases in this area. The agents capable of causing infectious diarrhea and the mechanisms responsible for disease pathogenesis are generally known. The causes of diarrhea in endemic areas include a wide variety of bacteria, viruses, and parasites. Most laboratories routinely screen for *Salmonella*, *Shigella*, and sometimes *Campylobacter*. Other bacteria, parasites, and viruses account for a significant percentage of diarrhea cases, but they frequently go no screened. So the true prevalence of these agents in developing countries is unknown [3]. Thus, it is necessary to determine a accurate picture of the situation in order to devise diarrheal disease-specific therapeutic measures and control strategies.

Unfortunately, the ability to detect an etiological agent is lacking in remote areas of many parts of the world, especially those where diarrheal disease is most prevalent and where most of the mortality occurs [4]. In Madagascar, microbiological methods for the clinical investigation of diarrheal diseases are usually restricted to the identification of conventional enteric bacteria because of a lack of resources. Accurate data on enteric pathogen prevalence for the whole country are not currently available or are very fragmented and almost useless for planning, implementing, and monitoring effective interventions [5]–[8].

In the present study, we determined the prevalence and the pathogenicity of bacterial, viral, and protozoal enteropathogens in diarrhea and non-diarrhea stools of children younger than 5 years of age in 14 different districts in Madagascar during a period of high diarrhea prevalence.

## Methods

### Setting and Enrollment

**Figure 1:**
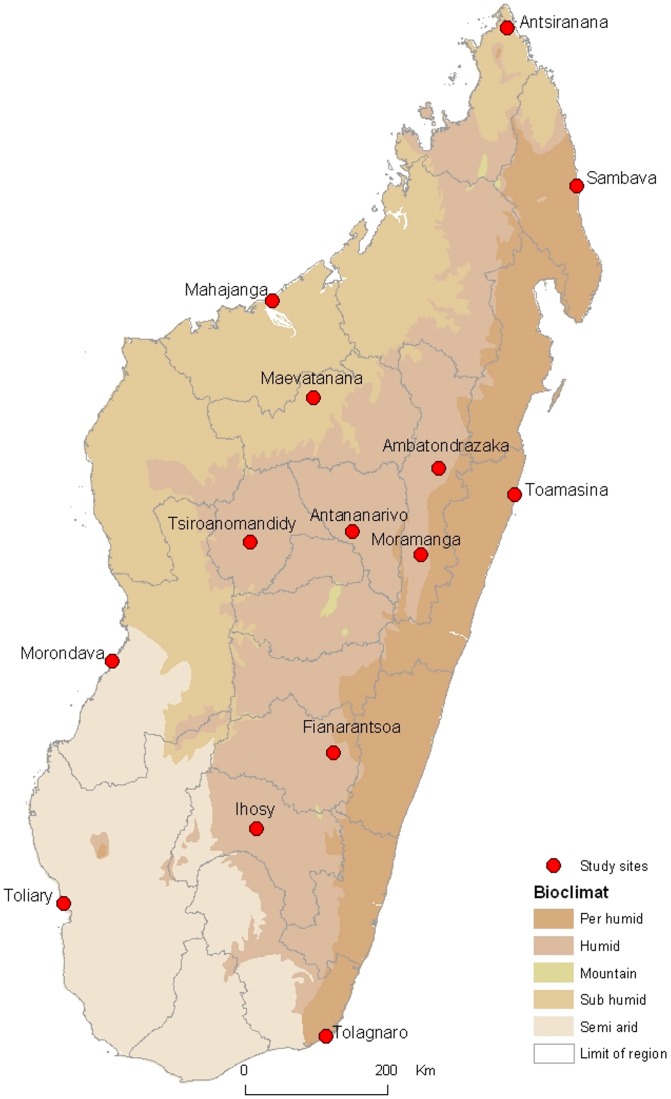
Location of study sites in Madagascar.

Madagascar, a large inter-tropical island with different climate profiles, is one of the poorest developing countries, experiencing recurrent politico-economical crises. In 2007, a syndromic sentinel epidemiological surveillance system was set up by the Pasteur Institute from Madagascar (IPM) throughout the country [9]. Among the 18 existing sites in 2008, the 14 first implemented since 2007 were selected for the study (Figure 1). The study was a cross-sectional survey of intestinal pathogens. It was conducted during rainy season periods from February 2008 to May 2009, for three consecutive weeks in each site. Official data from malagasy surveillance system showed that diarrheal syndromes were more frequent during the rainy season. For each site, routine surveillance data from the last 3 years highlighted the period with the greatest prevalence of diarrheal syndromes, allowing the schedule of the study to be better organized with two teams operating in the same time in two different sites.

The study was carried out in a community setting, not in hospitals or health centers. A mobile laboratory in a four-wheel-drive truck was used to perform the laboratory procedures in the best field conditions available as soon as the stools were emitted. First, different villages in each district were randomly selected. In each village, all children younger than 5 years of age with diarrheal syndrome were included in the diarrheal group. The non-diarrheal group was randomly selected from other healthy children younger than 5 years of age.

### Specimen and Data Collection

The participants provided clinical and epidemiological data and a fecal sample for the identification of various bacterial, viral, and protozoal enteropathogens. Case patients comprised children younger than 5 years of age with acute diarrhea without ongoing treatment. Diarrhea was defined as the emission of three or more unformed stools within a 24-h period. After stool emission, all case patients received treatment according to the Malagasy guidelines for diarrheal disease.

The non diarrheal subjects had no history of diarrhea in the previous week and were enrolled at the same time as case subjects. Information on demographic and historical clinical data were obtained from the parents of both cases and healthy subjects and recorded in questionnaires. No follow-up was performed after the initial recruitment of cases and healthy subjects.

### Laboratory Procedures

First of all, stool features were noted and stool specimens from diarrheic or non-diarrheic subjects were plated by field teams. Then, they were blindly analyzed for enteropathogens.

For detection of parasitic agents, a wet mount of each fresh fecal sample was prepared in a saline and iodine solution and observed under a light microscope for eggs and trophozoites of various parasites. The stool samples were further treated by the MIF concentration procedure, and a permanent slide was prepared for each sample. Samples that gave positive results with at least one method were considered positive.

The bacterial organisms, including *Salmonella* spp., *Shigella* spp., *Campylobacter* spp. and *Escherichia coli* (*E. coli*), were isolated using standard microbiological procedures. Stool samples were plated on Hektoen agar for the detection of *Salmonella* spp. and *Shigella* spp., on eosin methylene blue agar and UriSelect for the detection of *Escherichia Coli* and on Karmali agar for the detection of *Campylobacter* spp. All media were incubated at 37°C, with specific microaerophilic conditions for *Campylobacter* spp. (‘Campygen’, Oxoid England). *Campylobacter* spp. was confirmed using the hemagglutination test kit, ‘Campy dry spot’, from Oxoid, England, as recommended by the manufacturer.

Previously reported polymerase chain reaction (PCR) methods were used to screen isolates of *E. coli* for genes encoding virulence factors associated with diarrheagenic *E. coli* (DEC) [10].

For detection of rotavirus, adenovirus and astrovirus, two aliquots of a fresh stool specimen from each child were kept frozen at –80°C in cryo-vials (deidentified) until they were sent to the Institut Pasteur in Madagascar for viral analysis and biobanking. The presence of three enteric viral antigens, namely rotavirus, adenovirus, and astrovirus antigens, was investigated in the stool samples via the commercial immunoassays IDEIA Rotavirus, IDEIA Adenovirus, and IDEIA Astrovirus (Dako Ltd., Ely, United Kingdom), according to the manufacturer’s instructions.

### Ethical Issues

The national Ethics Committee of Madagascar approved the research protocol. Informed written consent was obtained from the next of kin, the legal and competent guardians on the behalf of the children involved in the study.

### Statistical Analysis

Statistical analysis was performed using the chi-square test or Fisher exact test. Two-tailed tests were used. Isolation frequencies were compared for each pathogen between cases and healthy subjects in order to analyze pathogenicity and between each pathogen in order to identify relation. To take in count the multicenter design of the study, logistic regression with the district as a random effects parameter was performed to study the relationship between status about diarrhea and independent variable (sex, age, clinical symptoms, pathogens…) selected according to bivariate analysis p-value less than 0.20. Odds ratios (OR) were calculated from the β coefficients. All statistical analyses were performed with a level of significance of 0.05.

## Results

During the study period, with no epidemic outbreak, 2,802 children were enrolled. Among them, 110 did not fulfill the following inclusion criteria: age <60 months (n = 15), no previous antimicrobial treatments, (n = 56, 21 (37.5%) had been receiving mebendazole and 12 (21.4%) cotrimoxazole), duration of diarrhea (n = 6), frequency of bowel movements (n = 10). Healthy subjects with abnormal or more than three stools in 24 h were also excluded (n = 25, including 1 with antimicrobial treatment and 1 aged more than 60 months).

Among the 2,692 children included in the analyses, 2,196 presented a diarrheal syndrome and 496 were asymptomatic controls. The sex ratio (male/female) was 1.10, it was respectively 1.12 in case group and 1.03 in healthy group (p-value = 0.38). The median age was 20.3 months (inter quartile range, IQR: 11.0–36.0); 19.0 months in the case group and 27.3 months in the non-diarrheal group (p<0.01).

In addition to diarrhea, the cases differed from the healthy subjects regarding other clinical signs (p<0.01): fever (13.0% versus 8.1%) and vomiting (10.7% versus 3.0%). No statistical difference was found concerning pulmonary symptoms (34.3% versus 31.0%, p = 0.17).

The diarrheal stools were loose in 1,417 cases (65%), associated with mucus in 111 cases and blood in 22 cases. They were watery in 562 cases (25%), associated with mucus in 100 cases and blood in 11 cases. They were mucosal but nor watery nor loose in 217 cases (10%), associated with bloody in 40 cases.

Various clinical symptoms were found in the diarrheal patients, the most frequent were: vomiting (n = 235, 10.7%), fever (n = 286, 13.0%), and dehydration (n = 171, 7.8%). The frequencies of these symptoms were statistically different between the two age groups analyzed (0–24 months and 24–60 months (Table1)).

**Table 1:** Clinical symptoms associated with the enteric pathogens isolated from diarrheic children in Madagascar 2008–2009.

	Cases detected	No. (%) positive					
		Fever		Vomiting		Dehydration	
**Age group**							
0–24 months	1286	209	(16.3)	182	(14.2)	115	(8.9)
24–60 months	910	77	(8.5)	53	(5.8)	56	(6.2)
p-value		<0.01		<0.01		0.02	
**Infections**							
Single	699	92	(13.2)	89	(12.7)	60	(8.6)
Multiple	501	56	(11.2)	47	(9.4)	34	(6.8)
Total	1200	148	(12.3)	136	(11.3)	94	(7.8)
No pathogendetected	996	138	(13.9)	99	(9.9)	77	(7.7)
**Agents**							
*Salmonella* spp.	32	5	(15.6)	4	(12.5)	9	(28.1)
*Shigella* spp.	38	7	(18.4)	4	(10.5)	7	(18.4)
*Campylobacter* spp.	209	35	(16.7)	27	(12.9)	20	(9.6)
*Campylobacter jejuni*	161	27	(16.8)	20	(12.4)	16	(9.9)
*Campylobacter Coli*	52	9	(17.3)	9	(17.3)	5	(9.6)
Adenovirus type 40/41	13	2	(15.4)	2	(15.4)	3	(23.1)
Rotavirus	112	19	(17.0)	53	(47.3)	11	(9.8)
Astrovirus	40	5	(12.5)	13	(32.5)	4	(10.0)
*Trichomonas intestinalis*	136	11	(8.1)	11	(8.1)	9	(6.6)
*Entamoeba hystolityca*	44	5	(11.4)	7	(15.9)	6	(13.6)
*Giardia lamblia*	276	19	(6.9)	11	(4.0)	19	(6.9)
Diarrheagenic *E. coli*	163	33	(20.2)	20	(12.3)	12	(7.4)

Intestinal microorganisms were isolated from 1,200 diarrheic patients (54.6%) and 228 healthy subjects (45.9%) (p<0.01).

At least one parasite was detected in 817 cases (37.2%) and 138 healthy subjects (27.8%, p<0.01). More than one parasite was present in 206 children and more often in patients (8.5%) than in healthy subjects (3.8%, p<0.01). Details of the parasitic isolates are presented in Table S1. *Giardia lamblia*, *Trichomonas intestinalis*, and *Entamoeba histolytica* were more prevalent (p<0.01) in patients with acute diarrhea (12.6%, 6.2%, 2.0%, respectively) than in healthy control subjects (7.7%, 2.0%, 0.4%, respectively) (table 2). No statistical difference was found between patients and healthy subjects for any other enteric parasites. The overall prevalence of enteric parasite infection was statistically different between the regions (p<0.01): *Giardia lamblia* was detected more often in the western region (Maevatanana, 26.2%, Mahajanga, 18.1%, Morondava, 20.2%), *Trichomonas intestinalis* was detected more often in the eastern region (Ambatondrazaka, 18.1%), as was *E. histolityca* (Moramanga, 5.0%, Ambatondrazaka, 4.0%) (Table S1). The multivariate analysis controlling for age and study sites as random effect showed that *Giardia* (OR = 1.9), *Trichomonas intestinalis* (OR = 4.2) were associated to diarrhea and to age group more than 24 months of age (respectively, OR = 3.5 and 3.0). In multivariate analysis, the relation between *Giardia* and diarrhea was higher in Maevatanana (OR = 3.9) and in Mahajanga (OR = 3.4).

**Table 2:** Enteric pathogens isolated from diarrheal and non diarrheal stools.

	Diarrheal stools				Non diarrheal stools				Univariate analysis		Multivariate analysis	
	tested	positive		%	tested	Positive		%	OR crude	95%CI	OR ajusted*	95%CI
Parasitic isolates	2196				496							
*Giardia lamblia*		276	(12.6)			38	(7.7)		1.7	[1.2–2.5]	1.5	[1.1–2.3]
*Trichomonas intestinalis*		136	(6.2)			10	(2.0)		3.2	[1.7–6.1]	3.5	[1.8–6.9]
*E. histolytica*		44	(2.0)			2	(0.4)		5.1	[1.2–20.9]	–	
Bacterial isolates	2196				2196							
*Salmonella* spp.		32	(1.5)			10	(2.0)		0.7	[0.4–1.5]	0.6	[0.3–1.3]
*Shigella* spp.		38	(1.7)			4	(0.8)		2.9	[0.9–9.4]	2.4	[0.7–8.1]
*Campylo.* spp.		209	(9.5)			47	(9.5)		1.0	[0.7–1.4]	0.9	[0.7–1.3]
*E. coli*	1286	175	(13.6)		220	32	(15.9)		0.9	[0.7–1.3]	0.7	[0.4–1.3]
Viral isolates												
*Rotavirus*	1667	112	(6.7)		364	15	(4.1)		1.7	[0.9–2.9]	1.3	[0.7–2.3]
*Adenovirus*	1594	98	(6.1)		347	13	(3.7)		1.7	[0.9–3.0]	1.7	[0.9–3.2]
*Astrovirus*	1003	40	(4.0)		239	6	(2.5)		1.6	[0.7–3.8]	1.5	[0.6–3.7]
										

* Random effect mutivariate analysis controled for study sites.

No significant difference were found among cases patients presenting with watery stools infected by *Giardia lamblia* (2.3%), *Trichomonas intestinalis* (5.1%) *or Entamoeba histolytica* (9.1%), or among cases patients presenting with bloody stools (respectively 23.3%, 31.6% and 36.4%).

Specimens from all 2,692 children were cultured (Table 2). At least one bacterial pathogen was identified in 329 patients (15.0%) and 71 healthy subjects (14.3%) (p = 0.70). *Campylobacter jejuni* were found in 161 cases with diarrhea (7.3%) and 36 healthy subjects (7.3%) and C*ampylobacter coli* in 52 cases (2.4%) and 11 healthy subjects (2.2%). *Salmonella* spp. were identified in 32 cases (1.6%) and 10 healthy subjects (2.0%). Only two were typed (1 *S. arizonae* and 1 *S. typhi*). *Shigella* spp. were identified in 38 patients (1.7%) and 4 healthy subjects (0.8%). *Shigella. flexneri* was detected most often (n = 28), although the prevalence was not statistically different between case group, (1.2% ) and healthy group (0.4%). *Shigella. dysenteriae* (n = 3) was only detected in one case group with diarrhea. Furthermore, four *S. sonnei* and three *S. boydii* isolates were detected in the case group and the healthy group (one of each serotype). More than one bacterial pathogen was found in 11 cases and 3 controls.

Diarrheagenic *Escherichia coli* were detected in 175 cases with diarrhea (13.6%) and 32 healthy subjects (14.2%) among the 1506 children who were aged 24 months or younger. Enterotoxinogenic *E. coli* (ETEC), Enteropathogenic *E. coli* (EPEC), Enteroaggregative *E. coli* (EAEC) and atypical EPEC (ATEC) were detected almost as often in the healthy subjects as in the cases (31.2% (10/32) vs. 37.2% (66/175), 21.8% (7/32) vs. 21.1% (37/175), 37.5% (12/32) vs. 36.0% (63/175), 18.8% (33/175) vs 25.0% (8/32) respectively) were detected more often in healthy subjects. Enteroinvasive *E. coli* (EIEC) and Shiga-like toxin-producing *E. coli* (STEC) were only detected in children with diarrhea (n = 4).

The random effect multivariate analysis controlling for age and study sites showed no significant difference in the prevalence of all bacterial isolates between the cases and healthy subjects. The overall prevalence of bacterial infection differed statistically according to the region (p<0.01): *Salmonella* were detected more often in the western region (Morondava, 8.1%, Mahajanga, 4.8%), *Shigella* were detected more often in the southern region (Taolagnaro, 3.8%, Toliara, 2.5%), and *Campylobacter* were found all over the country except for the central island and northwestern regions (Table S2).

The presence of rotavirus was tested by ELISA for 2,031 stool specimens (1667 cases and 364 controls): 127 (4.7%) (112 cases, 6.7%, and 15 healthy subjects, 4.1%) specimens were positive (Table 3). Among them, 119 (93.7%) were screened with PCR. The main genotypes were G9 (n = 57, 47.9%) and G1 (n = 34, 28.5%). Of the 1941 stool specimen tested (1594 cases and 347 healthy subjects), adenoviruses were found in 98 (6.1%) cases and 13 (3.7%) healthy subjects; among them, 59 stools (53.1%) were screened for adenovirus type 40 and 41, and these types were detected in 13 cases (26.5%) and 1 control (10.0%). Among the 1242 stool specimens analyzed (1003 cases and 239 healthy subjects), astroviruses were detected in 3.9% (n = 40) cases and 2.5% (n = 6) healthy subjects. The random effect multivariate analysis controlling for age and study sites showed no significant difference in the prevalence of rotavirus (OR = 1.3, 95%CI [0.7–2.4], adenovirus (OR = 1.7; 95%CI [0.9–3.1]) and astrovirus isolates (OR = 1.3; 95%CI [0.5–3.2]) between the case group and the healthy group. The prevalence of viral infection differed statistically according to the region (p<0.01): rotaviruses and adenoviruses were detected more often in Antananarivo, the main city (27.5%, 12.6%, respectively) and astroviruses were detected more often in Toamasina, the second main city of Madagascar, located on the eastern coast (15.8%) (Table S3).

Co-infections of bacteria and parasites were found in 166 children, viruses and bacteria in 34 children, viruses and parasites in 9 children and viruses, bacteria and parasites in 9 children.

The frequency of multiple infections was statistically higher (p<0.01) in the case group (22.8%, 501/2196) than in the healthy group (14.3%, 71/496) and among diarrheic children (p<0.01) in the age group of 24–60 months (34.2%, 311/910) compared with the age group 0–24 months (14.8%, 190/1286). No statistical difference was found according to gender.

The study of the relationship between pathogens showed that *Salmonella* infections were more often associated with *Campylobacter coli* (OR = 3.3, 95%CI:[1.1–11.1]), *Shigella* with *E.hystolitica* (OR = 4.8, 95%CI:[1.4–16.1]) and Giardia with Trichomonas intestinalis (OR = 1.7, 95%CI:[1.1–2.6]).

Multivariate analysis controlling for diarrheal status of the group and study sites (as random effect) showed a significant difference in the prevalence of *Campylobacter* and astroviruses isolates in age group less than 24 months of age (respectively OR = 1.5, 95%CI[1.1–2.0] and OR = 3.3, 95%CI[1.4–6.7]).

Furthermore, in diarrheic children, the frequency of clinical symptoms (vomiting, fever, or dehydration) was not statistically different according to the type of infection (Table 1). Table 1 shows the clinical symptoms associated with the enteric pathogens isolated from diarrheic children in Madagascar. *Salmonella* spp. and *Shigella* spp. were significantly associated with dehydration (OR = 4.8, OR = 2.7 respectively, p<0.01); diarrheagenic *E. coli* with fever (OR = 1.6, p = 0.01); rotaviruses and astroviruses with vomiting (OR = 10.8, OR = 4.2 respectively, p<0.01).

The random effect logistic regression analysis showed that vomiting (OR = 2.0, p<0.01), fever (OR = 1.5, p<0.01), and rotavirus (OR = 2.1, p<0.01) were significantly associated with diarrheic children under 24 months of age, although *Giardia lamblia* (OR = 0.3, p = 0.02) and *Trichomonas intestinalis* (OR = 0.3, p<0.01) were detected in this age group less often.

## Discussion

Few studies have been conducted to determine the etiology of childhood diarrhea throughout Madagascar during the most prevalent periods in relation to the rainy season. The results obtained in this study clearly show that many enteropathogenic bacteria, viruses, and parasites are endemic in Madagascar but the determination of the true etiological role of the enteric pathogens detected with diarrhea is difficult because enteric pathogens were also identified in apparently healthy children. Moreover, the study found more than one pathogen in numerous diarrheic subjects (55%) and, therefore, it was difficult to define which was causing the disease. Intestinal parasites were the predominant enteric pathogens in Madagascar during the rainy season. *Giardia lamblia* and *Ascaris lumbricoides* were the predominant species in the population studied, but a significant role in diarrhea was only found for *Giardia lamblia*, *Trichomonas intestinalis*, and *Entamoeba histolityca*. Furthermore, their distribution was not homogeneous in the different regions included in the study. These findings reflect the living conditions, lifestyle, and environmental conditions of the local population. Climate, food and water supplies, personal and community hygiene, sanitation, proximity to domestic and wild animals, and socioeconomic class are all implicated in the likelihood of exposure to intestinal parasites [11]. They are known to be associated with serious clinical diseases and mortality, and they are also known to cause malnutrition and impairments in physical development in children, affecting their growth and learning [11]. Thus, there is a great need to improve drinking water accessibility and to upgrade sanitation standards in Madagascar.

The prevalence of enteric bacterial pathogens was less important and was dominated by *Campylobacter* infections in the entire study population and by diarrheagenic *Escherichia coli* in the younger group. *Campylobacter* spp. are known to be important enteric pathogens, with *C. jejuni* usually being responsible for the majority of enteric *Campylobacter* infections [12]; this was also supported by the findings of the present study.

However, the proportions of cases and healthy subjects harboring *Campylobacter spp.* were similar and may be a sign of host factors such as immune status. Indeed, *Campylobacter* infection in developing countries usually appears to be restricted to children, with no strong pattern of seasonality and a higher incidence of infection complicated by a higher rate of asymptomatic carriage [13]. It has different clinical and epidemiological characteristics to those described for industrialized nations. Many of these differences are probably due to higher rates of exposure and infection earlier in life in developing countries, resulting in a different pattern of immunity [12]. What is interesting to highlight in our results is the higher frequency of campylobacter infection in diarrheic children under 2 years of age.

Data regarding the role of viral pathogens showed that rotavirus remained the main viral agent but its prevalence (6.7%) in diarrheic children was lower than expected compared to other African countries, with 13.4% in Libya [14] and 22.5% in Tunisia [15]. In the major published papers, the median duration of the studies was 12 months, with 25% of children having rotavirus infection [16]. The fact that our observational period in each sentinel site was limited to three weeks probably did not allow the detection of rotaviruses characterized by a year-round pattern [17]. Furthermore, this study was carried out in a community setting with active processes for catching diarrhea in children; in numerous other studies the children were included in a health-care setting and the majority of the published studies involved hospitalized patients or outpatients [16]. In regard to virological diagnosis, our study was limited by the absence of norovirus detection at this step of the work. Further laboratory tests are needed to estimate the prevalence of noroviruses in this two populations of children because little is known about the role of those viruses in cases of paediatric diarrhea in developing countries [18], [19].

The other limitations of our study were that the healthy subjects tended to be older than the case patients, a factor that we corrected by analyzing infections in two age groups. Also, it is possible that the mobile laboratory, although convenient and probably the only reliable tool for this study since the stools could be examined immediately, did not allow for the detection of some pathogens, a sensitivity identical to that obtained in built up laboratories. This might have modified the detection rates of some pathogens; however, if so, the bias would have been similar for both the cases and healthy subjects.

### Conclusions

Based on the results of this study, it was concluded that the high prevalence of the various enteropathogens among young children is a significant public health problem. Furthermore, our findings highlight the need to implement active surveillance programs for diarrheic stools that are not just based on hospitalized patients and to enhance laboratory capacities in Africa in order to support these programs.

## Supporting Information

Table S1 **Number and percentage of isolates of intestinal parasitic pathogens in children with diarrhea and non-diarrhea in Madagascar 2008–2009.**
(DOCX)

Table S2 **Number and percentage of isolates of bacterial pathogens in children with diarrhea and non-diarrhea in Madagascar 2008–2009.**
(DOCX)

Table S3 **Number and percentage of isolates of viral pathogens in children with diarrhea and non-diarrhea in Madagascar 2008–2009.**
(DOCX)
