# Supplementary material for: Case-Control Study of the Etiology of Infant Diarrheal Disease in 14 Districts in Madagascar
Source: PLoS One. 2012 Sep 17;7(9):e44533. doi: 10.1371/journal.pone.0044533 (PMC3444445; doi:10.1371/journal.pone.0044533)
Supplement: Table S1 — Number and percentage of isolates of intestinal parasitic pathogens in children with diarrhea and non-diarrhea in Madagascar 2008–2009. (DOCX) [file pone.0044533.s001.docx]

|  | **Case group** | | | | | | | | | | | | | | | |  | **Control group** | | | | | | | | | | | | | | | | |  |
| --- | --- | --- | --- | --- | --- | --- | --- | --- | --- | --- | --- | --- | --- | --- | --- | --- | --- | --- | --- | --- | --- | --- | --- | --- | --- | --- | --- | --- | --- | --- | --- | --- | --- | --- | --- |
|  |  | ***Giardia  lamblia*** | | ***Trichomonas intestinalis*** | | ***E. histolytica hist.*** | | ***Ascaris lumbricoïdes*** | | ***Trichuris  trichiura*** | | ***Hymenolepis  nana*** | | | ***Shistosoma mansoni*** | |  |  | ***Giardia  lamblia*** | | ***Trichomonas intestinalis*** | | | ***E. histolytica hist.*** | | ***Ascaris lumbricoïdes*** | | ***Trichuris  trichiura*** | | ***Hymenolepis  nana*** | | | ***Shistosoma mansoni*** | |  |
| ***Sites*** | **N** | ***n*** | ***(%)*** | ***n*** | ***(%)*** | ***n*** | ***(%)*** | ***n*** | ***(%)*** | ***n*** | ***(%)*** | ***n*** | ***(%)*** | ***n*** | | ***(%)*** |  | **N** | ***n*** | ***(%)*** | | ***n*** | ***(%)*** | ***n*** | ***(%)*** | ***n*** | ***(%)*** | ***n*** | ***(%)*** | | ***n*** | ***(%)*** | ***n*** | ***(%)*** |  |
|  |  |  |  |  |  |  |  |  |  |  |  |  |  |  | |  |  |  |  |  | |  |  |  |  |  |  |  |  | |  |  |  |  |  |
| Ambatondrazaka | 169 | 17 | (10.1) | 34 | (20.1) | 8 | (4.7) | 14 | (8.3) | 22 | (2.9) | 16 | (9.5) | 0 | | (0.0) |  | 30 | 3 | (10.0) | | 2 | (6.7) | 0 | (0.0) | 2 | (6.7) | 7 | (23.3) | | 1 | (3.3) | 0 | (0.0) | |
| Antananarivo | 161 | 3 | (1.9) | 3 | (1.9) | 7 | (4.3) | 21 | (13.0) | 1 | (0.6) | 0 | (0.0) | 0 | | (0.0) |  | 16 | 1 | (6.3) | | 0 | (0.0) | 0 | (0.0) | 2 | (12.5) | 0 | (0.0) | | 0 | (0.0) | 0 | (0.0) | |
| Antsiranana | 165 | 16 | (9.7) | 9 | (5.5) | 1 | (0.6) | 5 | (3.0) | 2 | (1.2) | 0 | (0.0) | 0 | | (0.0) |  | 39 | 3 | (7.7) | | 1 | (2.6) | 0 | (0.0) | 1 | (2.6) | 0 | (0.0) | | 0 | (0.0) | 0 | (0.0) | |
| Fianarantsoa | 154 | 2 | (1.3) | 0 | (0.0) | 2 | (1.3) | 84 | (54.5) | 7 | (4.5) | 3 | (1.9) | 4 | | (2.6) |  | 49 | 0 | (0.0) | | 0 | (0.0) | 0 | (0.0) | 19 | (38.8) | 0 | (0.0) | | 0 | (0.0) | 0 | (0.0) | |
| Ihosy | 135 | 20 | (14.8) | 4 | (3.0) | 2 | (1.5) | 1 | (0.7) | 0 | (0.0) | 20 | (14.8) | 14 | | (10.4) |  | 47 | 2 | (4.3) | | 1 | (2.1) | 0 | (0.0) | 1 | (2.1) | 0 | (0.0) | | 4 | (8.5) | 9 | (19.1) | |
| Maevatanana | 174 | 48 | (27.6) | 4 | (2.3) | 3 | (1.7) | 1 | (0.6) | 0 | (0.0) | 1 | (0.6) | 2 | | (1.1) |  | 28 | 5 | (17.9) | | 0 | (0.0) | 0 | (0.0) | 0 | (0.0) | 0 | (0.0) | | 0 | (0.0) | 0 | (0.0) | |
| Mahajanga | 138 | 29 | (21.0) | 16 | (11.6) | 7 | (5.1) | 5 | (3.6) | 3 | (2.2) | 7 | (5.1) | 0 | | (0.0) |  | 28 | 1 | (3.6) | | 2 | (7.1) | 2 | (7.1) | 1 | (3.6) | 0 | (0.0) | | 0 | (0.0) | 0 | (0.0) | |
| Moramanga | 175 | 15 | (8.6) | 10 | (5.7) | 10 | (5.7) | 24 | (13.7) | 5 | (2.9) | 0 | (0.0) | 0 | | (0.0) |  | 24 | 0 | (0.0) | | 0 | (0.0) | 0 | (0.0) | 17 | (70.8) | 1 | (4.2) | | 0 | (0.0) | 1 | (4.2) | |
| Morondava | 151 | 32 | (21.2) | 13 | (8.6) | 1 | (0.7) | 3 | (2.0) | 0 | (0.0) | 0 | (0.0) | 0 | | (0.0) |  | 47 | 8 | (17.0) | | 2 | (4.3) | 0 | (0.0) | 0 | (0.0) | 0 | (0.0) | | 1 | (2.1) | 0 | (0.0) | |
| Sambava | 134 | 5 | (3.7) | 2 | (1.9) | 0 | (0.0) | 8 | (6.0) | 4 | (3.0) | 0 | (0.0) | 0 | | (0.0) |  | 35 | 4 | (11.4) | | 0 | (0.0) | 0 | (0.0) | 0 | (0.0) | 4 | (11.4) | | 0 | (0.0) | 0 | (0.0) | |
| Toamasina | 159 | 24 | (15.1) | 7 | (4.4) | 0 | (0.0) | 26 | (16.4) | 29 | (18.2) | 2 | (1.3) | 1 | | (0.6) |  | 44 | 2 | (4.5) | | 0 | (0.0) | 0 | (0.0) | 3 | (6.8) | 7 | (15.9) | | 0 | (0.0) | 0 | (0.0) | |
| Tolagnaro | 176 | 34 | (19.3) | 12 | (6.8) | 0 | (0.0) | 20 | (11.4) | 30 | (17.0) | 2 | (1.1) | 0 | | (0.0) |  | 33 | 2 | (6.1) | | 0 | (0.0) | 0 | (0.0) | 1 | (3.0) | 0 | (0.0) | | 0 | (0.0) | 0 | (0.0) | |
| Toliara | 156 | 14 | (9.0) | 7 | (4.5) | 1 | (0.6) | 8 | (5.1) | 7 | (4.5) | 12 | (7.7) | 0 | | (0.0) |  | 48 | 3 | (6.3) | | 1 | (2.1) | 0 | (0.0) | 0 | (0.0) | 0 | (0.0) | | 2 | (4.2) | 0 | (0.0) | |
| Tsiroanomandidy | 149 | 17 | (11.4) | 15 | (10.1) | 2 | (1.3) | 8 | (5.4) | 0 | (0.0) | 9 | (6.4) | 0 | | (0.0) |  | 28 | 4 | (14.3) | | 1 | (3.6) | 0 | (0.0) | 1 | (3.6) | 0 | (0.0) | | 0 | (0.0) | 0 | (0.0) | |
|  |  |  |  |  |  |  |  |  |  |  |  |  |  |  | |  |  |  |  |  | |  |  |  |  |  |  |  |  | |  |  |  |  | |
| **TOTAL** | **2196** | **276** | **(12.6)** | **136** | **(6.2)** | **44** | **(2.0)** | **228** | **(10.4)** | **110** | **(5.0)** | **72** | **(3.3)** | **21** | | **(1.0)** |  | **496** | **38** | **(7.7)** | | **10** | **(2.0)** | **2** | **(0.4)** | **48** | **(9.7)** | **19** | **(3.8)** | | **8** | **(1.6)** | **10** | **(2.0)** | |
