# Supplementary material for: Case-Control Study of the Etiology of Infant Diarrheal Disease in 14 Districts in Madagascar
Source: PLoS One. 2012 Sep 17;7(9):e44533. doi: 10.1371/journal.pone.0044533 (PMC3444445; doi:10.1371/journal.pone.0044533)
Supplement: Table S2 — Number and percentage of isolates of bacterial pathogens in children with diarrhea and non-diarrhea in Madagascar 2008–2009. (DOCX) [file pone.0044533.s002.docx]

|  | ***Case group*** | | | | | | |  | ***& age<2years*** | | |  | ***Control group*** | | | | | | | |  | ***& age<2years*** | | |
| --- | --- | --- | --- | --- | --- | --- | --- | --- | --- | --- | --- | --- | --- | --- | --- | --- | --- | --- | --- | --- | --- | --- | --- | --- |
|  |  | ***Salmonella* spp*.*** | | ***Shigella* spp*.*** | | ***Campylo.* spp*.*** | | | ***E. coli*** | | |  |  | ***Salmonella* spp*.*** | | ***Shigella* spp*.*** | | | ***Campylo* spp*.*** | | | ***E. coli*** | | |
| **Districts** | N | n | % | n | % | n | % | | N | n | % |  | N | n | % | n | % | | n | % | | N | n | % |
| Ambatondrazaka | 169 | 4 | (2.4) | 0 | (0.0) | 24 | (14.2) | | 104 | 2 | (1.9) |  | 30 | 0 | (0.0) | 0 | | (0.0) | 2 | (6.7) | | 19 | 0 | (0.0) |
| Antananarivo | 161 | 0 | (0.0) | 4 | (2.5) | 5 | (3.1) | | 113 | 1 | (0.9) |  | 16 | 1 | (6.3) | 1 | | (6.3) | 0 | (0.0) | | 10 | 0 | (0.0) |
| Antsiranana | 165 | 3 | (1.8) | 2 | (1.2) | 15 | (9.1) | | 96 | 16 | (16.7) |  | 39 | 0 | (0.0) | 0 | | (0.0) | 4 | (10.3) | | 14 | 1 | (7.1) |
| Fianarantsoa | 154 | 0 | (0.0) | 1 | (0.6) | 6 | (3.9) | | 59 | 8 | (13.5) |  | 49 | 1 | (2.0) | 0 | | (0.0) | 2 | (4.1) | | 24 | 2 | (8.3) |
| Ihosy | 135 | 1 | (0.7) | 3 | (2.2) | 5 | (3.7) | | 81 | 30 | (37.0) |  | 47 | 1 | (2.1) | 0 | | (0.0) | 3 | (6.4) | | 11 | 3 | (27.3) |
| Maevatanana | 174 | 0 | (0.0) | 0 | (0.0) | 9 | (5.2) | | 99 | 26 | (26.3) |  | 28 | 0 | (0.0) | 0 | | (0.0) | 2 | (7.1) | | 8 | 4 | (50.0) |
| Mahajanga | 138 | 6 | (4.3) | 0 | (0.0) | 8 | (5.8) | | 81 | 1 | (1.2) |  | 28 | 2 | (7.1) | 1 | | (3.6) | 3 | (10.7) | | 14 | 0 | (0.0) |
| Moramanga | 175 | 1 | (0.6) | 3 | (1.7) | 36 | (20.6) | | 137 | 3 | (2.2) |  | 24 | 0 | (0.0) | 0 | | (0.0) | 5 | (20.8) | | 12 | 1 | (8.3) |
| Morondava | 151 | 12 | (7.9) | 7 | (4.6) | 26 | (17.2) | | 90 | 12 | (13.3) |  | 47 | 4 | (8.5) | 2 | | (4.3) | 5 | (10.6) | | 24 | 3 | (12.5) |
| Sambava | 134 | 1 | (0.7) | 2 | (1.5) | 15 | (11.2) | | 100 | 28 | (28.0) |  | 35 | 0 | (0.0) | 0 | | (0.0) | 0 | (0.0) | | 13 | 2 | (15.4) |
| Toamasina | 159 | 1 | (0.6) | 3 | (1.9) | 9 | (5.7) | | 70 | 22 | (31.4) |  | 44 | 0 | (0.0) | 0 | | (0.0) | 4 | (9.1) | | 21 | 8 | (38.1) |
| Tolagnaro | 176 | 2 | (1.1) | 8 | (4.5) | 24 | (13.6) | | 71 | 11 | (15.5) |  | 33 | 0 | (0.0) | 0 | | (0.0) | 7 | (21.2) | | 13 | 3 | (23.1) |
| Toliara | 156 | 1 | (0.6) | 5 | (3.2) | 22 | (14.1) | | 84 | 11 | (13.1) |  | 48 | 1 | (2.1) | 0 | | (0.0) | 9 | (18.8) | | 24 | 5 | (20.8) |
| Tsiroanomandidy | 149 | 0 | (0.0) | 0 | (0.0) | 5 | (3.4) | | 101 | 4 | (3.9) |  | 28 | 0 | (0.0) | 0 | | (0.0) | 1 | (3.6) | | 13 | 0 | (0.0) |
| **TOTAL** | **2196** | **32** | **(1.5)** | **38** | **(1.7)** | **209** | **(9.5)** | | **1286** | **175** | **(13.6)** |  | **496** | **10** | **(2.0)** | **4** | | **(0.8)** | **47** | **(9.5)** | | **220** | **32** | **(15.9)** |
