# Supplementary material for: Case-Control Study of the Etiology of Infant Diarrheal Disease in 14 Districts in Madagascar
Source: PLoS One. 2012 Sep 17;7(9):e44533. doi: 10.1371/journal.pone.0044533 (PMC3444445; doi:10.1371/journal.pone.0044533)
Supplement: Table S3 — Number and percentage of isolates of viral pathogens in children with diarrhea and non-diarrhea in Madagascar 2008–2009. (DOCX) [file pone.0044533.s003.docx]

|  | ***Case group*** | | | | | | | | | | |  | ***Control group*** | | | | | | | | |
| --- | --- | --- | --- | --- | --- | --- | --- | --- | --- | --- | --- | --- | --- | --- | --- | --- | --- | --- | --- | --- | --- |
|  |  | | | | | | | | | | |  |  | | | | | | | | |
|  |  | ***Rotavirus*** | | | ***Adenovirus*** | | | | ***Astrovirus*** | | |  | ***Rotavirus*** | | | ***Adenovirus*** | | | ***Astrovirus*** | | |
| **Districts** | **N** | n | | % | **N** | n | % | **N** | | n | % |  | **N** | n | % | **N** | n | % | **N** | n | % |
| Ambatondrazaka | 109 | 22 | (20.2) | | 109 | 10 | (9.2) | 98 | | 3 | (3.1) |  | 23 | 3 | (13.0) | 23 | 1 | (4.3) | 21 | 0 | (0.0) |
| Antananarivo | 142 | 42 | (29.6) | | 129 | 18 | (14.0) | 106 | | 7 | (6.6) |  | 14 | 1 | (7.1) | 13 | 0 | (0.0) | 12 | 0 | (0.0) |
| Antsiranana | 165 | 5 | (3.0) | | 164 | 8 | (4.9) | 69 | | 1 | (1.4) |  | 39 | 1 | (2.6) | 39 | 2 | (5.1) | 23 | 1 | (4.3) |
| Fianarantsoa | 95 | 5 | (5.3) | | 93 | 6 | (6.5) | 94 | | 2 | (2.1) |  | 27 | 0 | (0.0) | 26 | 3 | (11.5) | 27 | 1 | (3.7) |
| Ihosy | 96 | 5 | (5.2) | | 83 | 4 | (4.8) | 77 | | 4 | (5.2) |  | 41 | 1 | (2.4) | 36 | 0 | (0.0) | 33 | 0 | (0.0) |
| Maevatanana | 174 | 5 | (2.9) | | 162 | 5 | (3.1) | 115 | | 5 | (4.3) |  | 28 | 1 | (3.6) | 25 | 1 | (4.0) | 16 | 1 | (6.3) |
| Mahajanga | 134 | 3 | (2.2) | | 132 | 4 | (3.0) | 85 | | 2 | (2.4) |  | 28 | 0 | (0.0) | 28 | 0 | (0.0) | 15 | 0 | (0.0) |
| Moramanga | 167 | 0 | (0.0) | | 165 | 21 | (12.7) | 77 | | 2 | (2.6) |  | 21 | 0 | (0.0) | 19 | 3 | (15.8) | 13 | 0 | (0.0) |
| Morondava | 76 | 3 | (3.9) | | 75 | 3 | (4.0) | 69 | | 1 | (1.4) |  | 27 | 3 | (11.1) | 25 | 1 | (4.0) | 27 | 2 | (7.4) |
| Sambava | 132 | 13 | (9.8) | | 127 | 4 | (3.1) | 108 | | 6 | (5.6) |  | 34 | 4 | (11.8) | 32 | 0 | (0.0) | 29 | 0 | (0.0) |
| Toamasina | 159 | 0 | (0.0) | | 159 | 6 | (3.8) | 27 | | 6 | (22.2) |  | 44 | 0 | (0.0) | 44 | 0 | (0.0) | 11 | 0 | (0.0) |
| Tolagnaro | 65 | 5 | (7.7) | | 43 | 0 | (0.0) | 55 | | 1 | (1.8) |  | 9 | 0 | (0.0) | 8 | 0 | (0.0) | 9 | 0 | (0.0) |
| Toliara | 5 | 1 | (20.0) | | 5 | 0 | (0.0) | 5 | |  | (0.0) |  | 1 | 1 | (100.0) | 1 |  | (0.0) | 1 |  | (0.0) |
| Tsiroanomandidy | 148 | 3 | (2.0) | | 148 | 9 | (6.1) | 18 | | 0 | (0.0) |  | 28 | 0 | (0.0) | 28 | 2 | (7.1) | 2 | 1 | (50.0) |
|  |  |  |  | |  |  |  |  | |  |  |  |  |  |  |  |  |  |  |  |  |
| **TOTAL** | **1667** | **112** | **(6.7)** | | **1594** | **98** | **(6.1)** | **1003** | | **40** | **(4.0)** |  | **364** | **15** | **(4.1)** | **347** | **13** | **(3.7)** | **239** | **6** | **(2.5)** |
